# Assessing the impact of a novel dynamic stretching routine targeting myofascial chains for warm-up in trained adults

**DOI:** 10.3389/fspor.2026.1771756

**Published:** 2026-03-04

**Authors:** Luca Molinaro, Juri Taborri, Denis Pauletto, Valentina Guerra, Damiano Molinaro, Giovanni Sicari, Antonello Regina, Enrico Guerra, Stefano Rossi

**Affiliations:** 1 Department of Economics, Engineering, Society and Business Organization (DEIM), University of Tuscia, Viterbo, Italy; 2 ELAV, Città di Castello, Italy

**Keywords:** balance, fascia, heart rate, joint mobility, jumping, muscles, wearable sensors

## Abstract

**Background:**

Stretching is essential for maintaining overall health and is a key component of warm-up routines for well-trained individuals and athletes. The most commonly used stretching methods include static stretching (SS), dynamic stretching (DS), and ballistic stretching (BS). However, there is still an ongoing debate in the literature regarding which method is most effective, particularly with the growing interest in approaches that target myofascial chains rather than isolated muscle groups. In this context, the present study introduces a novel dynamic stretching protocol based on myofascial chains, designed to be incorporated into the warm-up routines of well-trained adults. The study aimed to evaluate the effects of this innovative protocol on mobility, balance, heart rate, lactate levels, and jumping performance.

**Methods:**

Nineteen well-trained men without sport-specific training were recruited for the experiment. The protocol consisted of three sessions, each differing in the activities performed between pre- and post-functional tests. On the first day, no activities (NA) were conducted. On the second and third days, participants were split into two groups, with one performing SS and the other following the novel DS routine. During each session, four functional tests were conducted: an incremental treadmill Run Test (RT), single-leg balance tests for both legs, repeated countermovement jumps, and joint mobility assessments.

**Results:**

The results indicated that both the innovative DS and SS protocols significantly improved joint mobility (*p*-values ranging from <0.001 to 0.049) and increased heart rate (*p*-values from <0.001 to 0.036). However, only DS led to a significant improvement in jump height (*p* = 0.026), whereas SS was associated with better balance, which was not observed in DS.

**Conclusions:**

These findings support the effectiveness of the novel dynamic stretching protocol, particularly as a warm-up strategy when explosive and reactive performance is required.

## 1 Introduction

Stretching refers to the deliberated and controlled elongation of muscles, tendons, and other soft tissues (1). It involves the movement of specific body parts or joints to their maximum extension or to a comfortable range with respect to their usual resting position (2). Stretching can be performed either actively, i.e., using the muscles themselves, or passively, i.e., with the assistance of external forces like gravity, equipment, or with the sup-port of another person (3). Stretching plays a crucial role in maintaining musculoskeletal health (4). Regular stretching exercises can improve flexibility by increasing the extensibility of muscles and connective tissues, this, in turn, enhances the range of motion (ROM) around joints, allowing for smoother and more efficient movements (2). In addition, stretching can help correct postural imbalances and address muscle imbalances caused by sedentary lifestyles or repetitive movements (5, 6). Furthermore, stretching exercises, particularly those incorporating deep breathing and mindful movements like yoga, have been found to promote relaxation and reduce stress levels (7).

Several different types of stretching techniques can be listed. Among others, the most widespread are static stretching (SS), dynamic stretching (DS), proprioceptive neuromuscular facilitation stretching (PNF) and ballistic stretching (BS) (8). SS is the most common type of stretching, involving holding a stretch in a comfortable position for a prolonged period, typically around 15–60 s. SS is often performed after physical activity or as a separate stretching routine (9) leading to an improvement of the circulation and an in-crease of the muscle temperature (10, 11). DS, instead, involves active movements allowing a full movement of the joint or muscle through its ROM. These movements are performed in a controlled manner, typically in a repetitive, rhythmic motions (12). DS has acute positive effects on muscle performance and injury prevention (12, 13). PNF stretching consists of a combination of stretching and contraction of muscles and it typically involves a partner or a stretching device to assist in the stretching process. PNF techniques involve contracting the muscle being stretched, then relaxing it while a partner applies a stretch. This method aims to enhance flexibility by using the body's neuromuscular reflexes (14). Finally, BS involves using bouncing or jerking movements to stretch muscles beyond their normal ROM (15). This type of stretching relies on the momentum generated by the movement to increase flexibility. However, it carries a higher risk of injury compared to other stretching methods and it is generally not recommended, particularly for individuals who are not highly trained or flexible (12).

Among the most common applications, stretching is an essential component of warm-up routines for athletes and individuals engaged in physical activities (16, 17). In fact, stretching allows to prepare the body for successive exercises by increasing blood flow to the muscles, improving neuromuscular coordination, and optimizing muscle activation. These benefits can lead to enhanced athletic performance, increased power output, and reduced risk of injuries preventing muscle strains, sprains, and other soft tissue injuries (18, 19).

However, there is still much debate about which is the best method of stretching to implement in the activity routine. A recent review by Lima et al. (20) on the acute effects of stretching on flexibility and performance pointed out how passive SS techniques elicit the greatest changes in flexibility in comparison with DS and BS, while inducing lower force and power output values when practiced over prolonged periods (>60 s for muscle group) and not combined with additional aerobic and sport-specific activities. At the same time passive SS may still be advantageous for highly flexible populations that focus on achieving high levels of ROM and improving technical sport tasks. Instead, the DS techniques may be the most effective to increase specific performance for athletes aiming to maintain moderate levels of ROM while avoiding performance decrements. The authors underline that there are many variables to keep under control to monitor output responses, such as duration, volume, intensity and frequency of stretching but also the population and the technology used for the analysis. Similar findings were highlighted in (8), where the authors evaluated the long-term effects on the ROM by varying the type and duration of the stretching. The study revealed an increment of the ROM from 11% for BS and 21% for SS and a variation from 15% to 20% based on the weekly frequency regardless the type of stretching. With regards to performance, Ryan et al. (21) examined the acute effects of different volumes of a dynamic stretching routine on vertical jump performance and flexibility in young adults, showing that a 6–12 min DS routine combined with 5 min of jogging increased jump height by 6% and sit and reach flexibility by 10%. Perrier et al. (22), showed a 3.9% DS and 2.6% SS increase in jump height compared to a warm-up protocol that did not include stretching, a nearly 10% increase in sit and reach flexibility for both SS and DS, and no change in reaction time, when gathered data from a cohort of recreationally active men. As regards the balance, in a recent review Behm et al. (23) highlighted how the acute effects of SS on balance is equivocal while DS is demonstrated to have acute beneficial effects on balance. In particular, longer-term stretch training can improve balance and thus help to reduce the incidence of falls.

By analyzing the literature, the studies focused on the evaluation of the effects due to different types of stretching implemented in their protocols exercises typically considered the single monoarticular or biarticular muscle group, whereas in recent years several studies focused the attention on the presence of myofascial chains along the body (24, 25). Fascia is a type of connective tissue that surrounds and envelops muscles, organs, and other structures. It consists of collagen and elastin fibers and has a role in providing support, maintaining structural integrity and transmitting mechanical forces (26). Myofascial chains are specific pathways or lines in the body where muscles and fascia are interrelated. These chains consist of a series of muscles and fascial structures that work together to produce movement and balance. These interconnected chains allow for the transmission of tension and forces throughout the body (27). From a study conducted by Stecco et al. (28) it emerged that the fascia is the primary target organ of stretching. To the best of authors knowledge, no warm-up protocol has been proposed in the literature that incorporates stretching exercises on myofascial chains addressed to well-trained adult subjects. There-fore, with this new perspective it is necessary to propose a new approach for the stretching exercise routine with a detailed execution protocol considering the myofascial chains. From this perspective, the aim of this paper is to propose a new stretching protocol for myofascial chains designed for well-trained adults to be performed as a warm-up routine. Through this scope, the effects induced by the innovative stretching protocol will be evaluated by using a sensor-based protocol. Finally, the stretching protocol will be performed both as static and dynamic procedure to compare the different modalities. The findings of the study can be used to adequately prepare the athletic condition of well-trained adults before physical activity in order to obtain the best benefits from the warm-up.

## 2 Material and methods

### 2.1 Participants

Nineteen well-trained male adults were involved in the study. Participants were classified as well-trained based on their regular engagement in structured physical activity for at least three years, with a training frequency of at least four sessions per week. This operational definition is consistent with commonly adopted criteria used in exercise science literature to describe trained but non-elite populations. Thus, as inclusion criteria, all the enrolled participants practice physical activity at different intensities since at least three years and for at least four times per week, having experience with different types of stretching. In particular, the subjects mainly practiced functional or resistance training in their weekly routine for a duration of each training of approximately 70–90 min, none of them practice sport-specific training. The participants' weight and height were measured with a professional scale (Seca 285, Seca ltd., Hamburg, Germany) typically used in clinics; the subjects stepped on the scale only with their underwear on and looked forward while standing. In addition, body composition values were also acquired to evaluate the health condition of the subjects. For this purpose, a bioelectrical analyzer (BIA 101 BIVA PRO, Akern Srl, Florence, Italy) at a single frequency of 50 kHz was used and the procedure has been conducted in accordance with standard protocols (29, 30). The measurements were carried out on the morning of the first day of the experimental protocol between 8 and 10 AM in a room with a temperature of 23–25 °C. After cleaning the skin, four low intrinsic impedance adhesive electrodes (Biatrodes Akern Srl, Florence, Italy) were placed on the right hand and right foot. The following parameters were automatically measured: (i) body resistance (R_c_) representing the ability of all biological structures to oppose the passage of electric current; (ii) body reactance (X_c_) indicating the ability of tissues to behave like capacitors that are therefore metabolically active; and (iii) the phase angle (Pha) that combines R_c_ and X_c_ providing an important predictor of health status. To avoid bias due to the inter-operator uncertainty, the same trained operator performed the BIA measurements. Subjects' characteristics are presented in Table 1. Individuals who had experienced injuries or undergone orthopedic surgery in the last ten were excluded from participation if the surgery can influence the health status of the subjects in terms of body's mobility and coordination. All participants were right-hand, whereas fifteen participants have the right leg dominant. The hand dominance has been reported by asking participants the hand used for writing; whereas the leg dominance has been obtained by asking participants to kick a ball (31). All participants were provided with comprehensive information regarding the purpose of the study, and their informed consent was obtained, adhering to the ethical guidelines established by the 1964 Declaration of Helsinki. The protocol has been approved on December 22nd, 2024 by the Ethical Committee of the University of Tuscia (n.22122024). All procedures were performed at the ELAV training and functional assessment center.

**Table 1:** Participants' details.

*N* = 19	Mean ± SD
Age (yr)	28 ± 6
Height (cm)	177.3 ± 5.9
Weight (kg)	75.7 ± 7.5
Years of training (n)	11.2 ± 3.9
Body Resistance (Ω)	434.9 ± 40.6
Body Reactance (Ω)	57.9 ± 5,7
Phase angle (°)	±0.5

### 2.2 Experimental protocol and setup

The experimental protocol consisted in performing three sessions: one session where the subjects did not perform any activity (NA), a session in which they performed static stretching (SS) and a session in which they performed dynamic stretching (DS). Functional tests were performed before (PRE) and after (POST) each session.

On day 1, before starting the data acquisition, a familiarization phase was performed in order to make subjects aware about the required experimental tasks and to avoid bias in the results due to learning process.

Without any kind of warm-up, the tests were performed every day on each group before NA, SS and DS activities to create the reference values. Subsequently, a one-hour break was performed and then NA, SS and DS were performed by each group, then the same tests were immediately performed with the same sequence.

On the first day, all subjects performed the functional tests without carrying out NA between PRE and POST. On days 2 and 3 the sample of subjects was randomly divided into two groups, one performed SS and the other DS, alternately on the two days. The randomization has been performed by reordering the names of the participants listed following an alphabetic order; after the reorder the participants associated with odd rows have been included in the first group and the remaining in the second group. To guarantee a complete washout after the execution of the functional tests, each experimental session was conducted at interval of two days. In addition, since the stretching protocol can be considered as warm-up, no further warm-up exercises were performed by the participants in the three experimental sessions.

**Figure 1:**
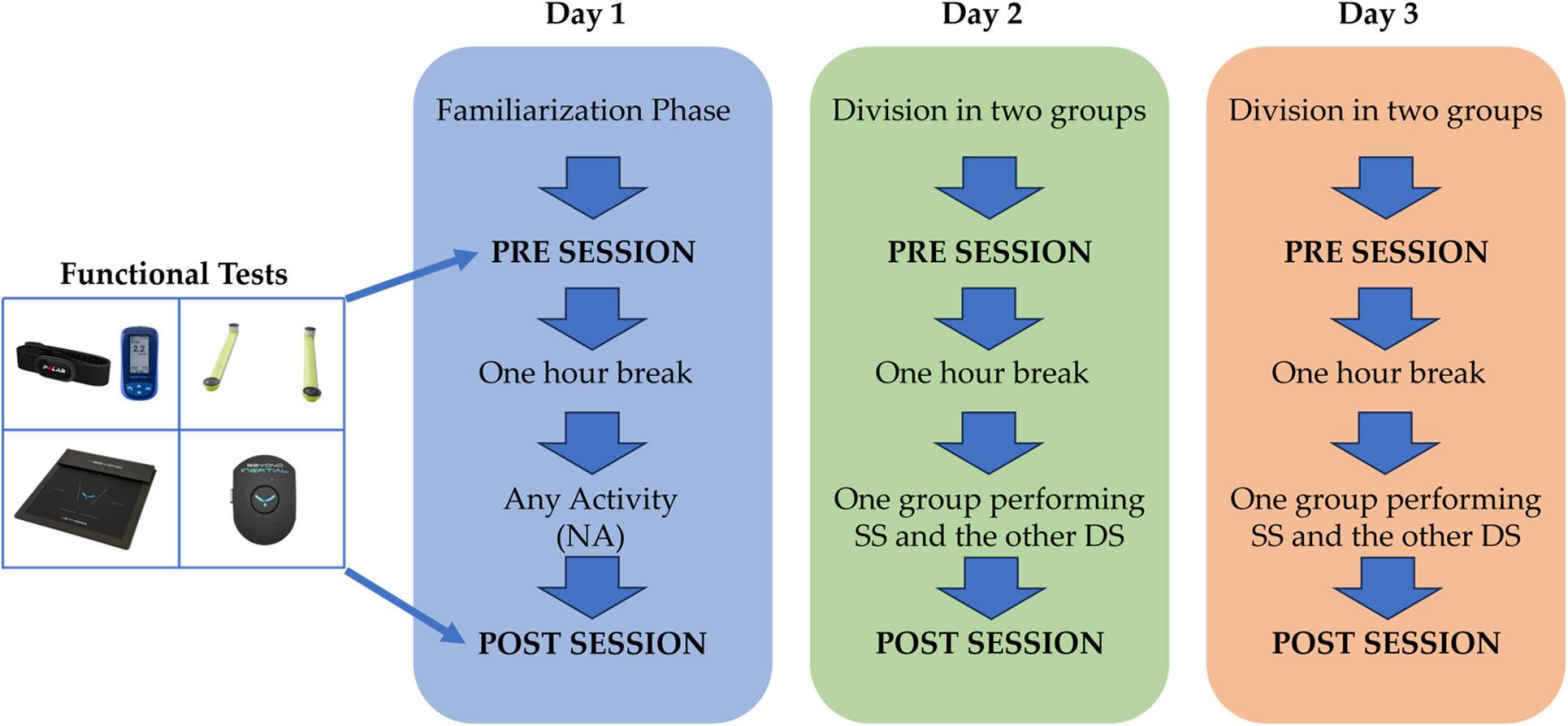
Block diagram of the experimental protocol and the devices used in the PRE and POST sessions, including sensor devices used for the functional tests and the groups division in the three days.

For each session, four functional tests were performed following the reported fixed sequence: (i) an incremental treadmill Run Test (RT) with heart rate monitoring by using the Polar H10 heart rate monitor (Polar Electro Oy, Kempele, Finland) and lactate evaluation using the lactacidimeter Lactate Scout 4 (SensLab GmbH, Leipzig, Germany); (ii) the analysis of the CoP displacements in monopodalic balance for both legs by using the Beyond Pressure 60 × 50 platform (Sensor Medica, Rome, Italy); (iii) the evaluation of the jump height during repeated countermovement jumps by using Optojump Next system (Microgate, Bozen, Italy); and (iv) the evaluation of joint mobility through the inertial sensor Beyond Inertial (Sensor Medica, Rome, Italy). The Beyond Inertial is equipped with the 9-axis motion tracking device ICM-20948 consisting in three-axis accelerometer, three-axis gyroscope and 3-axis compass. The ICM-20948 is one of the most widespread inertial units embedded in commercial inertial sensors, which are deeply validated in literature for motion analysis application (32, 33). A scheme of the experimental protocol and the devices used is shown in Figure 1.

#### 2.2.1 Static stretching

The static stretching protocol included four exercise stations. Stretching focused on four of the six main muscle chains: the superficial back line (SBL), the spiral line (SL), the lateral line (LL) and the front functional line (FFL) (25).

**Figure 2:**
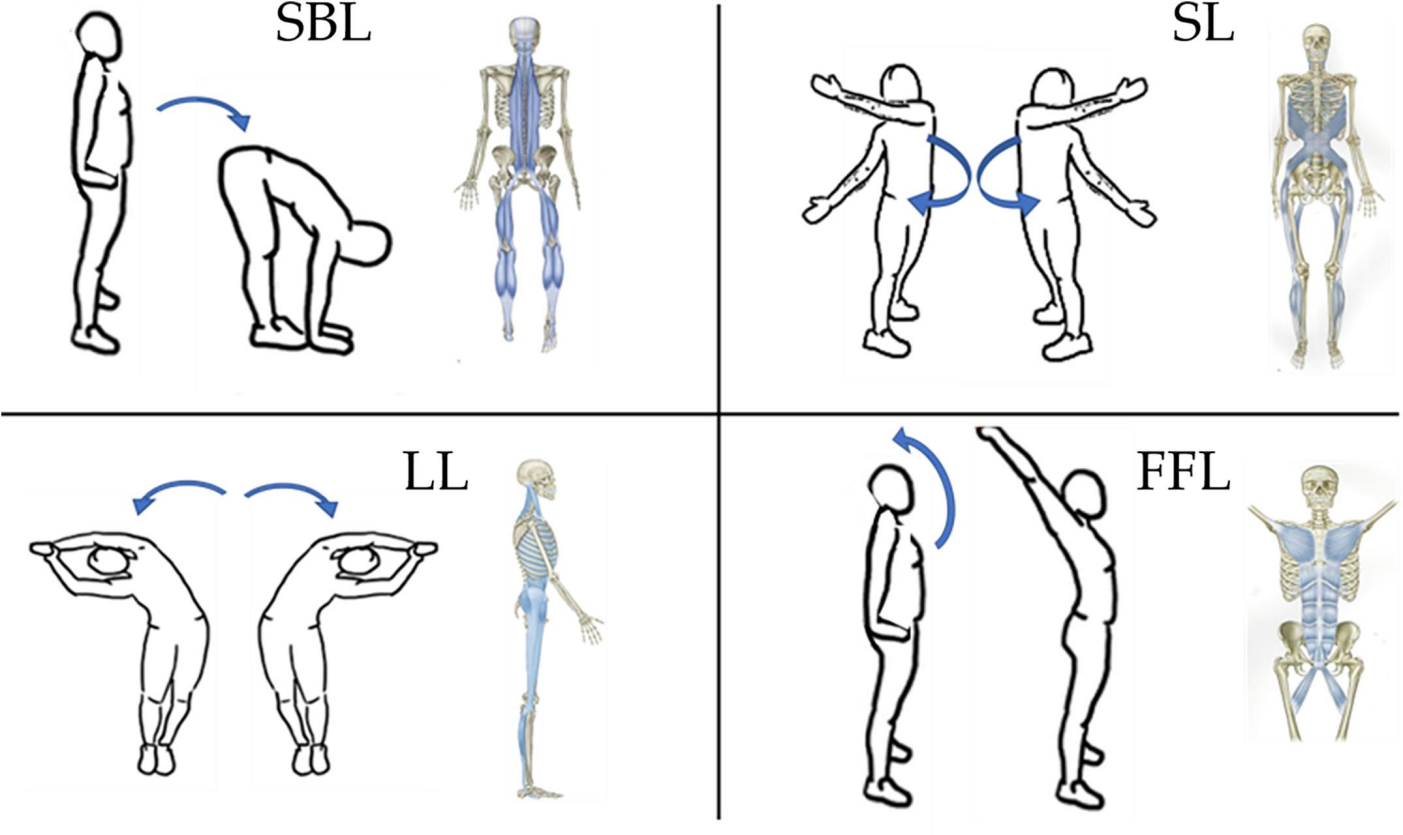
Stretching movements for the superficial back line (SBL), the spiral line (SL), the lateral line (LL) and the front functional line (FFL). In blue the myofascial chains solicited. Blue arrows indicate the direction of the movement between initial and final position.

In the SBL, from an upright position, the subject had to bend forward trying to touch the ground with the palms of the hands and avoiding bending the knees. In the SL, from the upright position the subject twisted the torso, keeping his feet firmly on the ground, bringing one arm back and the other in front of the torso in the same direction; after that, the movement was carried out in the opposite direction. In the LL, from the erect position and with the arms extended and joined above the head, the subject performed a lateral flexion keeping the feet firmly on the ground and closed each other, the movement was then carried out also in the other direction. In the FFL, from the erect position, the subject carried out an extension of the trunk bringing the pelvis slightly forward and the arms up and back extended above the head. All the above-described movements are shown in Figure 2. In all the movements, once the maximum stretching position was reached, the subject had to hold it for 30 s (34). After 30 s the participant moved to the next station, for the lateral line and the spiral line the movement was performed first on one side and then on the other. The circuit was repeated three times without taking any breaks for a total proto-col duration of about nine minutes.

#### 2.2.2 Dynamic stretching

The dynamic stretching protocol included the same four workstations shown for the static protocol shown in Figure 2 and the movements to be performed were also the same. Differently from the static stretching, the dynamic one provided, in the 30 s of work, that the subject had to quickly perform the stretching movement and once arrived in the position of maximum stretching, hold the position for only 1 s and then return to the initial position and, immediately, repeat the movement. For the LL, the movements were alternated between right and left and therefore the station became a single one lasting 1 min, the same for the SL. The circuit was repeated three times without taking any breaks for a total protocol duration of about nine minutes.

During all stretching exercises, both static and dynamic, the subjects were followed by trained operators who checked the correct execution of the movements.

#### 2.2.3 Joint mobility

Joint mobility tests were acquired with the Beyond Inertial sensor. The Beyond Inertial sensor system consists in a single Inertial Measurement Unit (IMU), with dimensions: 65 × 45 × 18 mm, and mass 28 g, that contains a triaxial accelerometer, gyroscope and magnetometer with full scale of ±16 g, ±2000°/s and ±4800 µT, respectively. The device has an internal 32-bit processor with floating point unit that processes the data and sends the results via Bluetooth 4.0 connection to the PC and stored using the Beyond Framework software (version 1.1.0.3). The sampling frequency was set at 500 Hz. The device can be fixed on a body segment using a dedicated semi-elastic belt equipped with a device-specific support to avoid relative movements during the execution of the task. Elastic belt guarantees subject comfort and reduces movement artifacts during task execution.

For the joint mobility assessment, the participants were asked to perform the same movements described in the stretching protocol with the sensor placed on the posterior trunk at the level of the T10 thoracic vertebra, exercising the maximum possible range at the preferred speed without compensating with other parts of the body. At the beginning of each test, the subject was asked to remain stationary in standing position in order to acquire a static phase of the inertial sensor and, successively, the subjects were asked to perform the gesture. A single repetition per each movement has been acquired.

In particular, the SL and LL movements were acquired separately for the right and left side (SL_RX, SL_LX, LL_RX and LL_LX) and the FFL movement was analysed by computing three indices, changing the positioning of the inertial sensor:

Analyzing trunk extension only (FFL_TE) with the sensor positioned as pre-viously described.
Analyzing shoulder flexion only (FFL_SF) by positioning the sensor on the arm at 15 cm from the center of rotation of the shoulder and performing only shoulder flexion in the sagittal plane without extending the trunk.
Analyzing complete movement of trunk extension and shoulder flexion (FFL_TESF) with the sensor in the same position described above.

**Figure 3:**
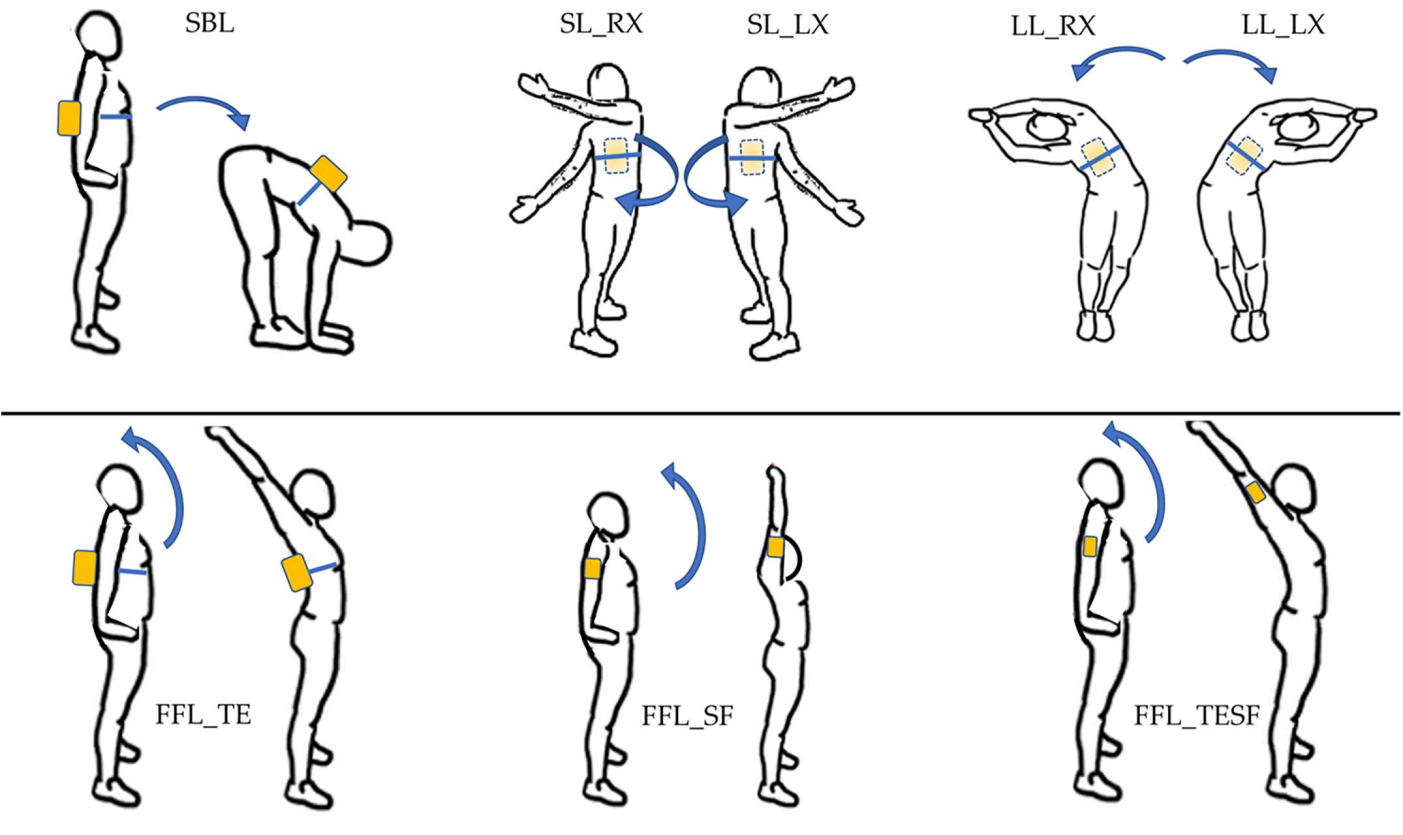
Joint mobility. Movements and positioning of the Beyond Inertial (yellow box). Blue arrows indicate the direction of the movement between initial and final position.

The movements analysed and the positioning of the sensor are shown in Figure 3.

#### 2.2.4 Treadmill run test (RT)

**Figure 4:**
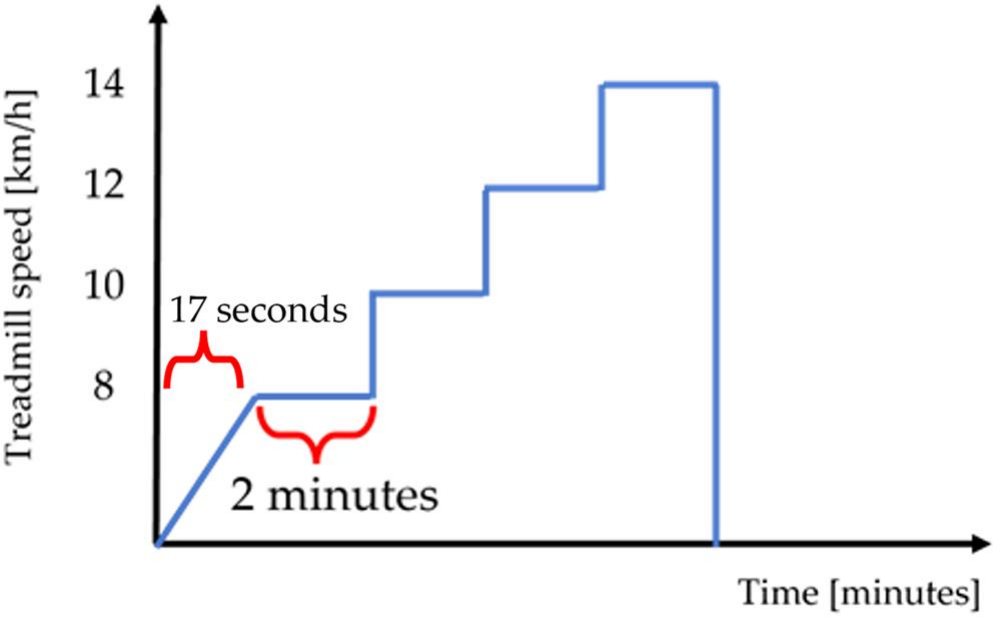
Time vs. treadmill speed during the RT.

The incremental RT was evaluated by monitoring the heart rate with the Polar H10 heart rate monitor whose validity has been demonstrated in several studies (35, 36). The heart rate monitor, placed with an adjustable elastic band on the subject's chest, acquires the heart rate at 1 kHz and transmits the data via bluetooth to the dedicated Polar Beat application (Polar Electro Oy, Kempele, Finland). During the test, the subject started on the treadmill Run 700 Excite LED (Technogym, Cesena, Italy) from a stationary position and the operator entered the speed of 8 km/h with a slope of 1% and the treadmill automatically reached the set speed in 17 s. At this point the subject ran for 2 min at 8 km/h and then he moved on to the next step with a speed of 10 km/h. The same steps were then performed, increasing the speed to 12 km/h and then to 14 km/h. The used treadmill has been utilized in previous research studies (37). A schematic of the RT is shown in Figure 4.

At the end of the test the lactate level [La-]b was acquired with the Lactate Scout 4 that is a capillary blood lactate detector using an enzymatic-amperometric method. The lactate in the sample is oxidized by the enzyme lactate oxidase and electrons are transferred from the enzyme to a functional electrode via an additional mediator. The resulting current corresponds to the lactate concentration of the sample. The device requires a 0.5 µL blood sample and has an analysis time of 15 s. Previous studies have investigated the accuracy and reliability of this device and it is now widely used (38, 39). The [La-]b analysis was per-formed by an experienced operator taking a blood sample from the left earlobe. Before each collection, the earlobe surface was disinfected and thoroughly dried. To avoid any unclean blood sampling the first drop has been eliminated. The sampling was performed 1 min after the end of RT, as in (40). It is worth noting that such an approach did not allow to measure the concentration curve and the lactate peak; however, our aim to have information at a certain time after the execution of the test in order to compare the effects due to different stretching protocols.

#### 2.2.5 Monopodalic balance (MB)

Beyond Pressure 60 × 50 platform was used to perform balance test. The platform (fullscale 150 N/cm^2^) consisted of 3.000 resistance sensors with a total are of 598 × 518 mm^2^, following a 50 × 60 matrix, in which each sensor has a sensitive area of 1 × 1 cm. Data was acquired with a sampling frequency of 25 Hz. The matrix is connected to the PC via a USB cable and works with the Beyond Framework software (version 1.1.0.3). The surface of the matrix is protected by using a conductive synthetic rubber Socaprene-based material. Metrological characteristics of the Beyond Pressure System was analyzed in a previous paper (41).

**Figure 5:**
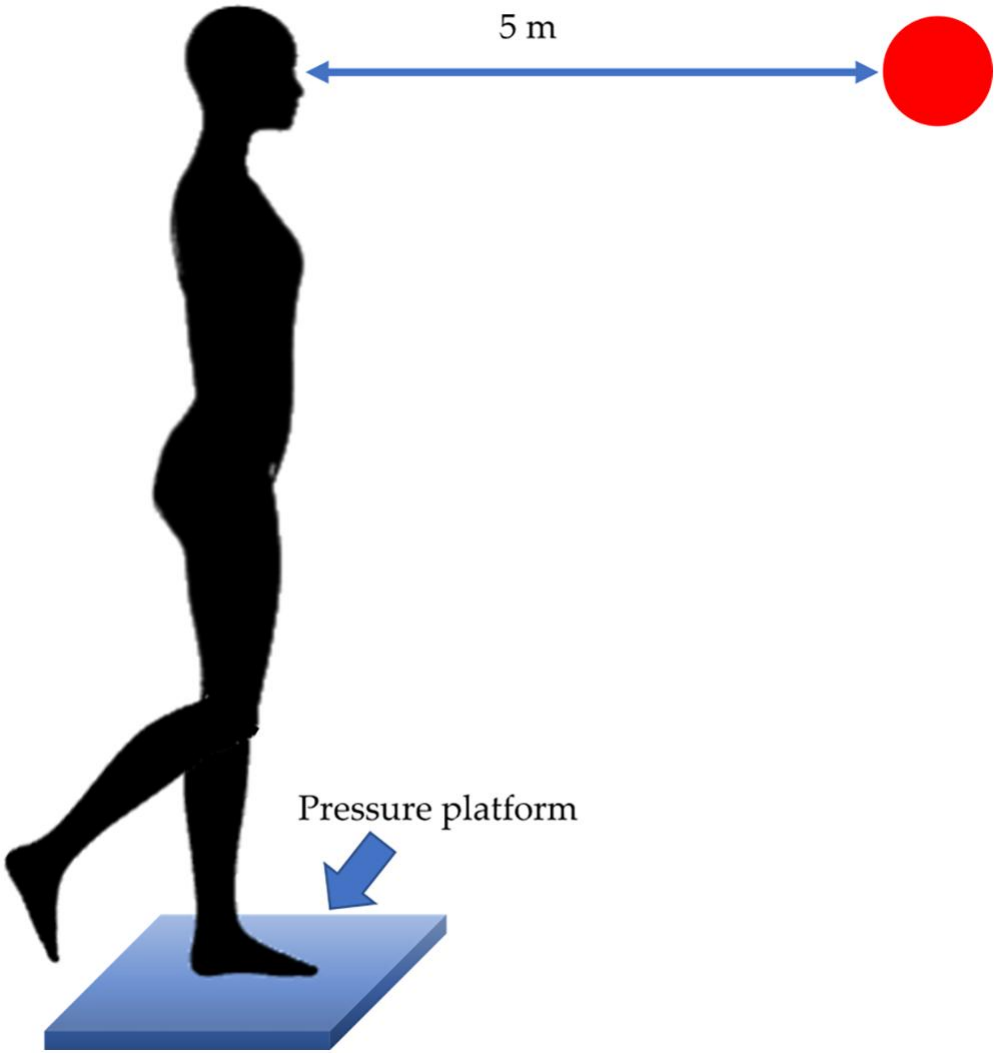
One-leg standing balance test. Positioning of the subject and the platform. Red dot represents the point to be fixed by the participants during the test.

For the evaluation of the equilibrium, a monopodalic balance test was performed (42). Subjects were instructed to lift only one leg off the ground by performing hip and knee flexion, while standing with arms relaxed at their sides. Subjects were asked to maintain this position for 30 s (Figure 5). During the test, subjects were also asked to fix a red point printed on the wall 5 m away. Tests were conducted with both lower limb sides without shoes, one time per each leg. Time between tests performed with the two lower limbs was set to one minute.

#### 2.2.6 Repeated countermovement jumps (RCJ)

**Figure 6:**
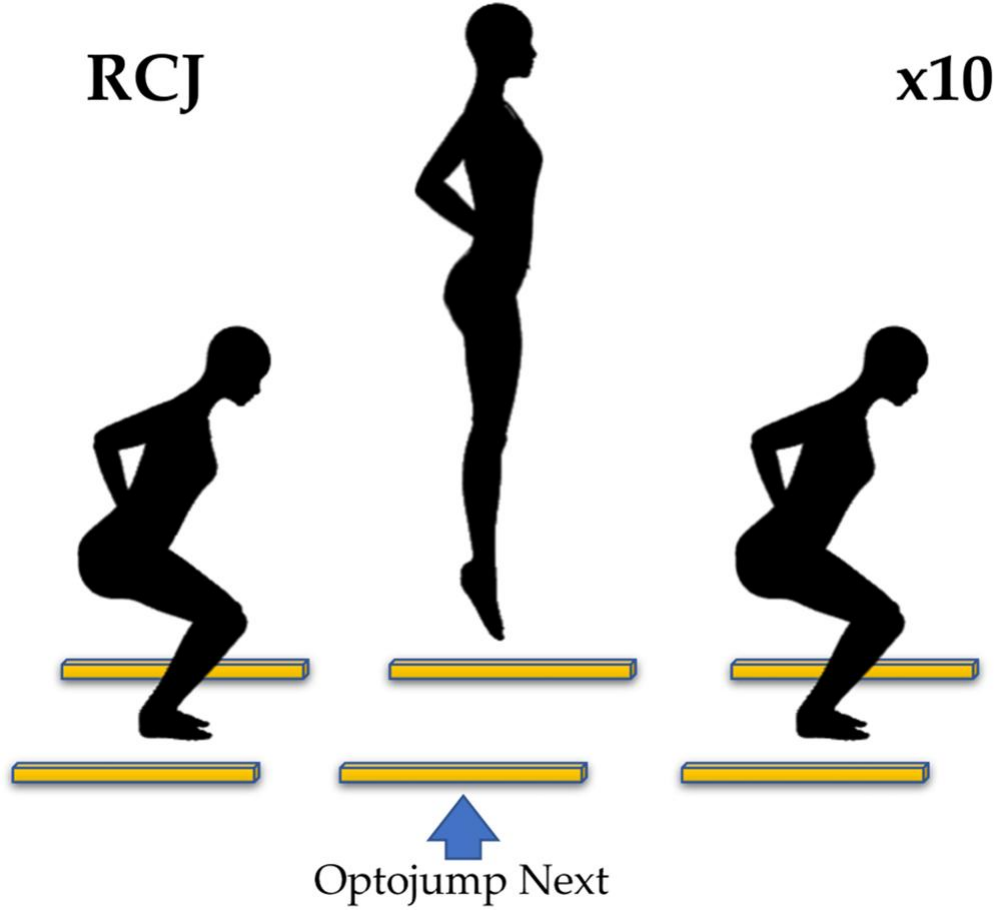
Repeated jumps. Positioning of the subject and the Optojump Next (yellow bars). X10 stands for the number of jumps.

The jump performance was assessed by asking subjects to perform repeated Countermovement Jumps (RCJ) and acquiring data by using the Optojump Next system, which consists of 1 m long transmitting and receiving bar, composed of ninety-six LED diodes, mounted above the ground. Optojump Next software version 1.12.23 (Microgate, Bozen, Italy) was used to acquire data. With this device it is possible to measure the flight and contact times with a resolution of 1 ms. The sampling frequency was set to 1 kHz. The Optojump Next system is widely considered for the analysis of flight and contact times during jumps (43). In the RCJ test, subjects are positioned centrally between the two optoelectronic bars placed 2 m apart and were asked to perform 10 maximal jumps from a standing position with the hands on the iliac crests (Figure 6). They were instructed to keep their hands on their bodies throughout the task and to jump as high as possible to minimize ground contact time without any restrictions of the knee angle during the squat phase (44). The test was performed with sports shoes on hard ground.

Tests were performed with a 2 min pause each other; the whole sequence was completed in approximately 20 min.

### 2.3 Data analysis

For each subject, the outputs of the five sensor systems were individually analysed according to the steps described below.

#### 2.3.1 Joint mobility

As regard joint mobility tests, the automatic re-alignment of the Beyond Inertial axes with the absolute reference system has been carried out by the Beyond Framework software with the data acquired during the initial static phase. The orientation of the sensor was computed by combining linear accelerations and angular velocities through a fusion algorithm based on the Mahony filter (45). We defined as θ the highest difference between the angle computed during the execution of the movement and the one gathered at the start of the task with the body segment in the rest position.

#### 2.3.2 Treadmill run test

The Heart Rate (HR) was analyzed by taking the instantaneous value shown by the Polar Beat application at the end of the run test per each tested speed, then HR8, HR10, HR12 and HR14 values were collected for each subject.

As regards the [La-]b, after taking the blood sample on the test strip it was inserted into the device until the measuring chamber was filled. After about 15 s the result of the [La-]b [mmol/L] appeared on the device display and was then recorded.

Regarding cardiovascular and metabolic responses, heart rate was interpreted as a functional indicator of warm-up effectiveness rather than as a comprehensive physiological outcome. Baseline heart rate values before the treadmill run test were not collected, as the experimental protocol was designed to focus on relative changes induced by stretching under standardized exercise conditions. While this choice limits a more detailed physiological interpretation, it is consistent with the engineering-driven and application-oriented nature of the study, which aimed to assess acute readiness for exercise rather than resting cardiovascular function. Similarly, blood lactate concentration was measured at a single post-test time point. This approach does not allow the characterization of the full lactate kinetics or peak response; however, it was intentionally adopted to minimize invasiveness, reduce testing time, and ensure protocol feasibility within a multi-sensor experimental framework. In this context, lactate was used as an indicator of the absence of fatigue-related effects rather than as a marker of metabolic performance.

#### 2.3.3 Monopodalic balance

The center of pressure (CoP) provided by the Beyond Pressure was used to measure the body balance. Following the relationships reported in (46), the CoP coordinates were computed as follows:

$$\left[\begin{array}{c} CoP_{x}\\ CoP_{y} \end{array}\right]=\frac{1}{\sum_{k}^{N}P_{k}*A_{k}}\left[\begin{array}{c} \overset{N}{\underset{k}{\sum }}P_{k}*A_{k}*x_{k}\\ \overset{N}{\underset{k}{\sum }}P_{k}*A_{k}*y_{k} \end{array}\right]$$

where (*x_k_, y_k_*) are the coordinates of the position of the *k*th sensor; *P_k_* is the pressure at the *k*th sensor and *A_k_* is the area of the *k*th sensor.

The CoPs of both the right and left stance were analyzed, leading to the computation of balance indices typically used for posturography analysis. In particular, following the equations reported in (47); we computed the ellipse area (EA), which is the area of 95% bivariate confidence ellipse estimated to contain 95% of the CoP, and the path length (PL), which is the total length of the path covered by the CoP.

#### 2.3.4 Repeated countermovement jumps

As regards the RCJ, the Optojump Next system provided the information related to the time instants in which the subject touched and detached the ground using the on-off status change of the LEDs of the optoelectronic bars. After the computation of the time flight, the system measures the jump height (JH) by applying the following equation:

$$JH=\frac{T_{v}^{2}*g}{8}$$

where *T_v_* is the time of flight and *g* is the gravity acceleration.

A total of eight jumps were analyzed discarding the first and the last jumps. Then, JH_A_ and JH_M_ were calculated as expression of the average height and maximum height of the eight jumps, respectively.

### 2.4 Statistical analysis

The statistical power of the analysis was computed with software G*Power (48). The outcomes of the power statistical analysis showed a mean power value of about 87% with a medium effect size (0.5). The analysis was designed to assess within-condition PRE–POST effects, in accordance with the study aim of evaluating the physiological and functional impact of the proposed stretching protocol rather than performing direct comparisons between different warm-up modalities. Data were first tested for normality with the Shapiro–Wilk test. To analyze any significant differences due to the effects on the above-mentioned physical and physiological parameters of performing NA, SS and DS between the PRE and POST sessions, a paired-samples *T*-test was used independently on all the computed parameters and for the three different tasks. Statistical significance was set at *p* *<* 0.05 for all the performed tests. A Bonferroni correction was applied within each condition and outcome domain to control the family-wise error rate associated with multiple PRE–POST comparisons. When the significance has been found, the effect size has been computed following the Cohen's criterion computing the parameter *d*, which can range from 0 to infinite, where greater d, higher the effect size. The effect size was then classified as: (i) very low, when *d* was lower or equal to 0.20; (ii) low when *d* was ranged from 0.20 to 0.50; (iii) medium when *d* ranged from 0.50 to 0.80; and (iv) large in case of *d* greater than 0.80, accord to (49). The SPSS 17.0 software application for Windows was used for statistical analysis.

## 3 Results

The mean values and standard deviations of all NA, SS and DS parameters for the PRE and POST sessions are reported in Table 2.

**Table 2:** Mean values (standard deviations) of all NA, SS and DS parameters for the PRE and POST sessions. Light grey cells indicate a medium effect size, whereas the dark grey a large effect size.

Parameters	NA		SS		DS	
	PRE	POST	PRE	POST	PRE	POST
$\theta_{SBL}$ [°]	142.3 (15.7)	144.9 (16.0)	146.4 (15.5)	151.7 (13.7)*	146.0 (16.2)	151.9 (12.2)*
$\theta_{SL\_RX}$ [°]	86.2 (14.9)	88.5 (20.0)	102.3 (15.4)	108.8 (16.3)*	99.6 (20.7)	103.9 (13.5)
$\theta_{SL\_LX}$ [°]	87.5 (16.7)	89.9 (17.0)	98.6 (16.1)	103.9 (16.1)*	95.1 (16.8)	99.8 (12.5)
$\theta_{LL\_RX}$ [°]	53.8 (9.0)	55.3 (7.7)	58.5 (8.9)	62.3 (11.8)*	56.9 (7.5)	61.2 (7.0)*
$\theta_{LL\;LX}$ [°]	53.6 (8.6)	55.7 (8.4)	60.9 (8.8)	65.0 (10.4)*	57.7 (9.9)	61.9 (7.9)*
$\theta_{FFL\_TE}$ [°]	37.4 (12.3)	38.9 (11.9)	39.1 (7.0)	42.9 (9.3)*	39.6 (9.4)	41.8 (11.1)
$\theta_{FFL\_SF}$ [°]	177.3 (13.5)	177.3 (12.8)	174.9 (10.0)	178.8 (9.8)*	177.3 (11.2)	179.4 (10.4)
$\theta_{FFL\_TESF}$ [°]	208.9 (11.6)	209.2 (126)	207.7 (11.8)	211.1 (12.7)	207.7 (11.0)	209.6 (11.0)
HR_8_ [bpm]	138.6 (13.0)	140.6 (12.3)	129.3 (8.9)	131.7 (10.2)*	130.8 (9.6)	133.3 (9.6)*
HR_10_ [bpm]	154.8 (12.7)	155.5 (12.2)	144.7 (10.9)	147.1 (10.4)*	146.6 (9.9)	148.5 (10.0)*
HR_12_ [bpm]	169.4 (10.7)	170.0 (10.9)	159.6 (11.5)	162.7 (10.8)*	161.6 (10.0)	165.8 (10.2)*
HR_14_ [bpm]	179.2 (9.7)	180.3 (10.3)	172.6 (10.5)	175.7 (10.5)*	173.5 (10.3)	177.7 (10.1)*
[La^−^]_b_ [mmol/L]	5.8 (2.5)	5.4 (2.2)	5.1 (2.4)	5.1 (1.9)	5.0 (2.7)	5.3 (2.1)
EA_DX_ [mm^2^]	4.7 (2.8)	4.5 (4.0)	3.4 (1.5)	2.6 (1.2)*	3.6 (2.1)	3.6 (3.4)
EA_SX_ [mm^2^]	4.8 (3.0)	4.8 (2.7)	3.4 (1.9)	3.3 (2.6)	2.9 (1.1)	3.8 (2.3)
PL_DX_ [mm]	76.8 (18.8)	70.4 (25.5)	64.5 (14.5)	56.9 (14.5)*	60.9 (14.9)	63.6 (18.3)
PL_SX_ [mm]	78.7 (24.9)	71.0 (26.0)	58.5 (8.3)	51.4 (17.3)	56.6 (13.9)	60.6 (17.7)
JH_A_ [cm]	31.6 (4.9)	32.6 (5.4)	32.3 (5.9)	32.9 (6.5)	32.6 (5.4)	33.8 (5.7)*
JH_M_ [cm]	33.9 (4.4)	35.8 (5.4)	35.2 (5.9)	35.8 (6.6)	35.0 (6.0)	36.6 (5.7)*

NA, any activity; SS, static stretching; DS, dynamic stretching; *θ*_SBL_, superficial back line angle; *θ*_SL RX_, right spiral line angle; *θ*_SL LX_, left spiral line angle; *θ*_LL RX_, right lateral line angle; *θ*_LL LX_, left lateral line angle; *θ*_FFL TE_, trunk extension angle; *θ*_FFL SF_, shoulder flexion angle; *θ*_FFL TESF_, trunk extension plus shoulder flexion angle; HR_8_, heart rate value at the end of the 8 km/h step; HR_10_, heart rate value at the end of the 10 km/h step; HR_12_, heart rate value at the end of the 12 km/h step; HR_14_, heart rate value at the end of the 14 km/h step; [La^−^]_b_, lactate level; EA_DX_, ellipse area of the right limb; EA_SX_, ellipse area of the left limb; PL_DX_, path length of the right limb; PL_SX_, path length of the left limb; JH_A_, average height of the eight jumps; JH_M_, maximum height of the eight jumps.

* Asterisk represents statistical differences between PRE and POST.

By analyzing the results, regarding NA no statistical differences were found. In all the cases *p*-value ranged between 0.188 and 0.986.

In the SS, significant differences emerged for all the parameters of the joint mobility with the exception of *θ*_(FFL_TESF) (*p*-value = 0.175). Significant differences also emerged for HR8, HR10, HR12, HR14 with *p*-value = 0.020, *p*-value = 0.028, *p*-value <0.001 and *p*-value <0.001 respectively. Considering MB, significant differences emerged regards EADX and PLDX (*p*-value = 0.037 and *p*-value = 0.030, respectively). No significant differences emerged in the other parameters analyzed with *p*-value ranged between 0.329 and 0.918.

In the DS, significant differences emerged for *θ*_SBL (*p*-value = 0.049), *θ*_(LL_RX) (*p*-value = 0.014), and *θ*_(LL_LX) (*p*-value = 0.048), in other cases of the joint mobility *p*-value ranged between 0.131 and 0.209. Significant differences also emerged for HR8, HR10, HR12, HR14 with *p*-value = 0.036, *p*-value = 0.020, *p*-value <0.001 and *p*-value <0.001 respectively. Moving to RCJ, significant differences emerged regards JHA and JHM (*p*-value = 0.049 and *p*-value = 0.026, respectively). No significant differences emerged in the other parameters analyzed with *p*-value ranged between 0.110 and 0.804.

## 4 Discussions

Through the aim to measure the effects induced by an innovative myofascial chain-based dynamic stretching protocol, an experimental protocol involving nineteen well-trained adults were performed. Data gathered from the used experimental setup allowed to quantify the effects in terms of heart rate, lactate level, joint mobility, equilibrium and jump ability. The reported values associated with Pha, RC and Xc are similar to those shown by Campa et al. (50), allowing us to confirm the health status of the enrolled sub-jects and the possibility to consider them as well-trained.

As expected, results reveal as the absence of stretching routine does not lead to any differences in terms of performance when repeating functional tests.

By focusing on the mobility, the results allow affirming that the here proposed DS routine permit to achieve one of the main goals of a warm-up session, which is the in-crease of joint flexibility (8). This result is in line with the analysis of other static and dynamic protocols, already proposed in literature (20–22). The increase of the mobility can be ascribed to the increase of muscle temperature due to the active and repetitive contractions of the muscle (51); in fact, it is demonstrated as an increase of the temperature leads to a reduction of the viscous resistance with a consecutive improvements of tissue flexibility (52). However, it should be highlighted that the increase of the mobility can be observed only for some of the mobility indices when focusing on DS, conversely all the indices are significantly greater in case of SS. This finding is likely due to the timing of the DS with respect to the SS since during the dynamic stretching less time is spent in the stretched position, reducing the relaxation effect from viscoelastic stress.

By moving to HR and [La-]b, it can be noted as the behavior of SS and DS is the same, leading a significant increase of the heart rate in all the examined conditions, whereas no changes are observed in the lactate level. The increase of the heart rate is generally in contrast with the literature, in which it was affirmed as stretching routine significantly reduces arterial stiffness and, consequently, the heart rate (11). However, it should be underlines as these findings are related to subjects with pathologies, where the stretching leads to a structural modification of the blood vessels and to a reduction of the vascular stiffness with a consecutive decrease of the flow resistance (10). Considering that the aim of a warm-up for well-trained people is generally the increase of the heart rate, it is possible to validate the suitability of the innovative dynamic stretching protocol. The increase of the heart rate in well-trained adults is justified by the necessity of the heart to increase the cardiac output in order to compensate the restricted blood flow due to the mechanical obstruction generated by the muscle contraction (53). In addition, it is worthy noticing that the increase in terms of heart rate associated with DS is about 25% greater than the in-crease due to the static stretching protocol. This difference could be ascribed to the active and rhythmic contraction of the muscles during the DS (12). The absence of differences in terms of lactate level suggests that the implemented functional test does not induce fatigue-based effects, as well the stretching routine, which can be then used as warm-up where fatigue has not to be faced by people.

As concerns the equilibrium, greater performance in terms of balance control was also found for the SS when considering the dominant leg; whereas no effects due to the DS was showed. These results are in line with the findings reported in (23), since it was shown as the benefits of dynamic stretching on balance control can be only observed in long-term training, whereas few repetitions of SS can lead to an improvement of dynamic balance in the execution of single leg balance task. In fact, acute bouts of SS have been demonstrated as capable to reduce musculotendinous unit stiffness allowing the unit to react with sufficient force and speed to centre of pressure movements (54). In addition, the influence of limb dominance on monopodalic balance was also affirmed in (55). Additionally, it is worth noticing, that with the exception of (23), previous literature often reports neutral or positive acute effects of dynamic stretching on balance (56), these discrepancies may be explained by task specificity and methodological differences. In the present study, balance was assessed through a static monopodalic stance, a task that predominantly relies on sensory integration and fine postural control rather than on reactive or dynamic stabilization mechanisms. Acute bouts of static stretching may reduce musculotendinous stiffness, thereby enhancing proprioceptive sensitivity and facilitating the regulation of center-of-pressure oscillations during static tasks. Conversely, dynamic stretching is known to acutely increase neuromuscular activation and excitability, which may be advantageous for dynamic or reactive balance tasks but less effective in purely static postural conditions. Furthermore, the observed improvements were mainly associated with the dominant limb, supporting the influence of limb dominance on monopodalic balance performance.

Finally, the analysis of the results associated with the jump performance is in line with the literature in which the positive effect of DS was demonstrated with respect to the SS (21). Similar results have been obtained also by (22). It is useful to under-line that such a result has been obtained when performing consecutive jumps, allowing to enlarge the literature results that have been, instead, obtained only on single jump. The improvement in muscular performance in achieving greater height during jumps can be ascribed to the elevated muscle temperature, which leads to an enhancement of metabolic reaction and to an increase of the nerve conduction velocity of action potentials (57). The improvement in jump performance observed after dynamic stretching is also consistent with recent studies reporting acute enhancements in explosive and reactive performance following warm-up or activation-based protocols in trained adults (58). These immediate benefits are commonly attributed to increased muscle temperature, enhanced motor unit recruitment, and improved neural drive. Recent investigations on acute warm-up and activation strategies have shown similar effects on jump performance, suggesting that the observed improvements may reflect general neuromuscular activation mechanisms rather than stretching-specific effects. Within this framework, the proposed myofascial chain–based dynamic stretching protocol appears effective in eliciting such acute responses, while its multi-joint and chain-oriented structure may further contribute to movement coordination and force transmission during repeated countermovement jumps.

For sake of completeness, this study reports some limitations. The cohort of subjects only included male well-trained adults; thus, gender effects were not considered. In addition, for a complete evaluation of professional sport, different types of athletes should be tested. As concerning the experimental protocol, the results associated with the lactate only represent the value at a certain time after the execution of the test, without providing in-formation related to the lactate's peak, as well as the absence of a baseline of the heart rate before the execution of the run test can lead to a bias in the results. Finally, although several PRE–POST differences reached statistical significance, findings associated with small-to-moderate effect sizes should be interpreted with caution.

## 5 Conclusions

The selection of the appropriate stretching routine can influence the physical and physiological performance of well-trained adults. Our results show that the here proposed innovative myofascial chain-based dynamic stretching routine is suitable for being integrated into a warm-up of well-trained adults. In fact, an increase of heart rate without facing fatigue was found, as well an improvement of the joint mobility for the majority of the analyzed movements. In addition, the proposed protocol revealed itself as able to increase the jump performance with respect to the static stretching, whereas no effects on the equilibrium were observed. These findings can be exploited for coaching to use the innovative stretching protocol, especially when the explosive-reactive performance of well-trained male are required. In particular, sports in which jump and sprint activities are crucial can benefit from the reported findings, as for example football, basketball, etc. Future studies should investigate whether similar effects can be observed in female populations and in athletes involved in sport-specific training, in order to improve the external validity of the proposed protocol and its generalizability.

## Data Availability

For data, contact the corresponding author.
